# Editors' Note

**DOI:** 10.5195/ijt.2020.6343

**Published:** 2020-12-08

**Authors:** Ellen R. Cohn, Jana Cason

**Affiliations:** 1 IJT Editor; 2 Senior Associate Editor

The *International Journal of Telerehabilitation* (IJT) is a biannual journal dedicated to advancing telerehabilitation by disseminating information about current research and practices. The journal is indexed by PubMed and Scopus.

## ISSUE OVERVIEW

This issue was produced in the days of the unrelenting 2020 COVID-19 pandemic. As would be expected for an e-journal, these unusual circumstances did not alter IJT's editorial and production processes, as IJT journal editors and publishing staff have always worked remotely. What was a departure from issues produced pre-COVID19, was the volume of new submissions, nearly a five-fold increase. This quickly required an enlarged pool of reviewers. Fortunately, our professional networks generously assisted, and IJT was able to quickly recruit many more expert reviewers.

We cannot over-state the contributions that volunteer reviewers make to a journal, its authors, and to current and future readers. Discerning reviewers are essential to the quality control of every journal. The purpose of an IJT review is not just to advise the editors on whether to publish an article. Most importantly, reviewers dig deep inside an article and if needed, to advise authors how to elevate the quality of their work. We are most grateful to an assiduous group of new and returning anonymous reviewers, about whose efforts our authors routinely offer appreciation. One experienced scholar recently wrote: “Again, we want to pass on our sincere thank you for considering this submission and what a reviewer this is! They are exceptional and have provided great feedback.”

The current issue of the multi-disciplinary *International Journal of Telerehabilitation* (IJT) features impactful research that spans rehabilitation disciplines and medical diagnoses. Among the eleven curated articles, three detail how telerehabilitation is strategically deployed in parts of three underserved regions: Brazil, Croatia, and Nigeria. IJT reviewers and editors are keenly aware that cultural and socioeconomic variables influence how telerehabilitation is most appropriately defined and creatively administered in different cultures. We are confident that IJT readers will absorb the content in these articles with cultural humility and recognize that the parameters of telerehabilitation are best locally determined. We invite even more work from non-US countries.

## CALL FOR SUBMISSIONS

We cordially invite submissions to the Spring 2021 issue in mid-January 2021. IJT accepts original research, case studies, viewpoints, technology reviews, book reviews, and country reports that detail the status of telerehabilitation.

